# Cilnidipine: An L- and N-Type Blocker of Calcium Channels Ameliorating Renal Damage in Experimental Hypertensive Rats

**DOI:** 10.7759/cureus.81404

**Published:** 2025-03-29

**Authors:** Gouher Banu Shaikh, Kusal K Das

**Affiliations:** 1 Department of Physiology, BLDE (Deemed to be University) Shri B. M. Patil Medical College, Hospital and Research Center, Vijayapura, IND

**Keywords:** ace, angiotensin ii, creatinine clearance, hypertensive rats, l and n type calcium channel blockers, proteinuria, ras, renal damage

## Abstract

Background: Cilnidipine is both an L-type and N-type calcium channel blocker (CCB). Cilnidipine suppresses hyperactivity in the renin-angiotensin system and sympathetic nervous system by blocking N-type calcium channels. We hypothesized that through its N-type calcium channel blockade, cilnidipine may reduce proteinuria and improve creatinine clearance in hypertensive rats.

Aim of the study: The current study was done to show the renal protective effect of cilnidipine on chronic hypertensive rats. Our primary objective is to develop a hypertensive rat model by giving L-NAME (NG-nitro-L-Arginine Methyl Ester Hydrochloride) and 4% sodium chloride (4% NaCl). High salt was given along with L-NAME because the combination of L-NAME and high salt intake leads to a synergistic effect on blood pressure. L-NAME impairs NO production, and high salt intake exacerbates this effect by promoting vasoconstriction and fluid retention. Our secondary objective is to evaluate the kidney damage measures, including recording of proteinuria, creatinine clearance, and urinary angiotensin II (Ang II) levels in hypertensive rats with or without cilnidipine treatment.

Methods: Six groups of male Albino Wister rats (with six rats in each group) were created by a simple randomization technique. Rats were obtained from the animal house of our institution. Group 1 (control) received vehicle treatment; cilnidipine (2mg/kg/day by oral gavage) was given to Group 2; L-NAME (40mg/kg/day by oral gavage) was given to Group 3; Group 4 received L-NAME plus cilnidipine; Group 5 received L-NAME plus 4% NaCl (mixed with diet) treatment; Group 6 received L-NAME, 4% NaCl and cilnidipine. Creatinine excretion and urinary protein were assessed in a 24-hour urine sample. Serum urea and creatinine levels were also measured. Relative expression of serum and renal tissue ACE (angiotensin-converting enzyme) protein was done by Western blotting. Quantitative estimation of urinary and serum Ang II levels was done by enzyme-linked immunosorbent assay (ELISA). Kidney histopathological analysis was carried out.

Results: In the renal tissue homogenate and serum of L-NAME and salt-induced hypertensive rats, we found elevated ACE and Ang II levels. We also observed a significant increase in proteinuria (4.65±0.29) compared to control (1.56±0.044) and a decrease in creatinine clearance (0.06±0.0019) compared to control (0.078±0.0044) in hypertensive rats. We observed that in treatment with cilnidipine (groups 4 and 6 rats), there were significant improvements in creatinine clearance (0.077±0.0027) (p < 0.05) and a significant decrease in proteinuria (3.38±0.24) (*p *< 0*.*05). In rats treated with cilnidipine, we also observed significantly decreased levels of Ang II in the urine and serum (*p *< 0*.*05) and a significantly decreased expression of ACE in the renal tissue and serum.

Conclusion: These results showed that in hypertensive experimental rats, cilnidipine, apart from its hypotensive actions, decreases proteinuria and urinary creatinine and Ang II levels. These actions of cilnidipine may be because of blocking N-type calcium channels. Therefore, cilnidipine dual L/N type CCB may act as a renoprotective drug and decrease glomerular damage. Further mechanistic studies using selective N-type channel blockers or knockout mice are needed to prove the findings.

## Introduction

Cilnidipine is a fourth-generation dihydropyridine that acts slowly and for a long time. It is a blocker of both L-type and N-type calcium channels. L-type calcium channels are located in the smooth muscle of blood vessels, whereas N-type calcium channels are found in presynaptic nerve terminals. Apart from its antihypertensive actions, cilnidipine could also reduce oxidative stress and suppress the renin-angiotensin system (RAS) in blood vessels [1].

Long-term renal failure is a result of many morphological and quantitative changes in the kidney due to persistent hypertension, including the loss of glomeruli. The main medications used to treat chronic nephropathy are blockers of angiotensin receptors and angiotensin-converting enzyme (ACE) inhibitors [2].

Reducing hypertensive renal injury should be the primary goal of treatment, as the kidneys are the main organs affected by chronic hypertension. An important factor in the increasing damage to the kidneys is sympathetic hyperactivity. Therapies that reduce sympathetic overactivity may be beneficial for the long-term renal disease. Therefore, in rats with hypertensive renal damage, cilnidipine, a dual L/N type CCB, may be more helpful than an L-type calcium channel blocker alone. Toba et al., Konno et al., and Fan et al. also reported that cilnidipine treatment, compared to amlodipine, signiﬁcantly reduced the renal Ang II levels and podocyte injury in experimental hypertensive rats. They suggested that the renal protective mechanism of cilnidipine may be because of RAS suppression [3-5].

By decreasing the sympathetic nervous system, cilnidipine prevents glomerular hypertension through its N-channel blocking activity. In addition, cilnidipine reduces oxidative stress by suppressing RAS and protects podocytes. Numerous renal disorders may occur as a result of an excess of reactive oxygen radicals. Cilnidipine exhibits a stronger reno-protective effect than amlodipine because of its anti-oxidative property [6].

It has been demonstrated that the rat hypertension model deficient in nitric oxide is a valuable tool for investigating the onset and management of renal lesions similar to those observed in human hypertension [7]. High salt in nitric oxide-deficient rats increases the production of superoxide and hence contributes to the development of a salt-sensitive hypertensive rat model with renal injury [8].

Although previous studies have shown the effectiveness of L-type calcium channel blockers in hypertension, the role of N-type blockade in renal protection remains underexplored.

Therefore, we investigated how cilnidipine protected the kidneys in hypertensive rats treated with L-NAME and salt.

## Materials and methods

Thirty-six male Albino Wister rats (160 and 200 grams in weight) aged 60-70 days were procured from an institutional animal house. All the animals were allowed to acclimatize to the laboratory conditions for one week before initiating the experimental protocol. The animals were maintained at 22-24 degrees Celsius room temperature and exposed to a 12-hour light and12 12-hour dark cycle with food and water made available ad libitum. All experiments are carried out in compliance with the guidelines set forth by the Committee for Purpose and Control and Supervision of Experiments on Animals (CCSEA) by the Indian government.

The sample size was calculated using the resource equation approach [9].

Sample size=DF/k +1 

where DF: Degree of freedom; k: no. of groups, which is 6 (present study)

Minimum n/group= 10/k +1= 10/6+1=2.6=3

Maximum n/group=20/k +1=20/6+1= 4.33=5

For the proposed study, 3 to 5 animals per group are required. In other words, a total of 18 to 30 rats are required to keep the DF within the range of 10 to 20.

In the present study, n=6 rats/group, i.e., 36 rats for six groups were taken.

Animal intervention

Rats were divided into six groups randomly after a week of acclimatization. Group 1 (Control) received a vehicle of 0.5% Na CMC (sodium carboxymethyl cellulose). Group 2 received cilnidipine in 0.5% Na CMC, in a dose of 2.0mg/kg/day by oral gavage [10]. Group 3 received L-NAME (40mg/kg body weight) along with distilled water by oral gavage. Group 4 received L-NAME and Cilnidipine. Group 5 received 4% NaCl in diet and L-NAME. Group 6 received L-NAME, cilnidipine, and 4% NaCl. Every medication is administered orally every morning for a period of 28 days.

L-NAME and salt-induced hypertensive rat model

We prepared a hypertensive rat model by administering L-NAME (40mg/kg/day in distilled water by oral gavage) and 4% NaCl mixed with diet. The combination of L-NAME and high salt intake leads to a synergistic effect on blood pressure. L-NAME impairs NO production, and high salt intake exacerbates this effect by promoting vasoconstriction and fluid retention. As NO levels are reduced by L-NAME, the ability of blood vessels to dilate in response to stimuli is impaired, contributing to sustained high blood pressure. High salt intake disrupts normal kidney function by promoting sodium retention and activating the renin-angiotensin-aldosterone system (RAAS), both of which contribute to increased blood pressure [11].

Recording of blood pressure

Systolic blood pressure and diastolic blood pressure of each rat were recorded weekly by an NIBP (non-invasive tail cuff) method under consistent conditions. Before measuring the blood pressure, the animals were put in a restrainer for 10-20 minutes/day for one week for acclimatization and minimizing stress. The tail is kept at an appropriate temperature using a heating pad for 30 minutes to improve the detection of tail artery pulsations. In the NIBP method, a tail cuff sensor (Bio Pac MP 100: PC Windows-based animal electrophysiology system) was used to record blood pressure, and Bio Pac Student Lab 4.1 software (BIOPAC System Inc., Goleta, USA) was used to analyze all the parameters. Three recordings were done for each rat, and the mean of the three measurements was considered. The following formula is used to calculate mean arterial pressure (MAP): MAP = Diastolic blood pressure+1/3 pulse pressure [12].

Biochemical parameters

Serum urea and creatinine were estimated by drawing blood from the supraorbital plexus. About 5ml of blood was collected from each rat, once just before the sacrifice of the rats. The serum was separated and kept at -20°C. A VITROS 5.1/FS automated dry chemistry analyzer (Ortho-Clinical Diagnostics, Inc., Rochester, USA) was used to measure the amounts of urea and creatinine in the serum.

Collection of urine

Each rat was housed in metabolic cages for one week for acclimatization, and then samples of urine were collected every 24 hours from 10:00 am in the morning to the next day at 10:00 am to measure the excretion of creatinine and protein in the urine. All urine samples were centrifuged to remove sediments [13]. An auto analyzer (VITROS 5.1/FS chemistry system) was used to measure the 24-hour protein concentration. Creatinine clearance was calculated by using the following formula:

Creatinine clearance = (urine creatinine (mg/ml) ×urine volume/day in mL/ 1440 min /serum creatinine (mg/ml)/both kidney weight in gm [14].

Qualitative expression of ACE by western blotting

ACE protein estimation was done in serum and kidney tissue homogenates. Serum ACE and kidney tissue ACE protein expression was done by using primary antibodies, Cat No-ACE (2E2): sc-23908, dilution 1:500, and secondary antibodies, goat anti-mouse IgG1: HRP -Cat No-STAR132P.

Equal amounts of protein (20μg) were loaded into the wells of a midi (13.3x8.7cm) format sodium dodecyl sulfate polyacrylamide gel electrophoresis (SDS-PAGE), along with molecular weight markers. Protein separation by gel electrophoresis was done by using a voltage of 120V for one hour. Protein was transferred from the gel to the membrane by using 100 V for 90 minutes. Primary antibodies were incubated overnight, and the membrane was kept at 4 degree centigrade temperature on a rotator. The blot was rinsed five times for 5 min with Tris-buffered saline with Tween 20 (TBST) and incubated in the HRP-conjugated secondary antibody solution for one hour at room temperature. Then the blot was rinsed five times for 5 min with TBST. A chemiluminescent substrate was applied to the blot according to the manufacturer’s recommendation. The chemiluminescent signals were captured using a charge-coupled device camera-based imager. Image analysis software was used to read the band intensity of the target proteins. Housekeeping protein transferrin and beta actin were used for serum and tissue, respectively.

Quantitative estimation of angiotensin II (Ang II) in serum and urine by ELISA

An Angiotensin II ELISA Kit (Cat No-k11-0656) was used to estimate levels of urinary and serum Ang II levels. 96-well plates pre-coated with the purified anti-Ang II antibody were used. The anti-Ang II biotin-labeled antibodies were used as detection antibodies. The standards, test samples, and horseradish peroxidase (HRP) conjugate were added to the wells, mixed and incubated, and then unbound conjugates were washed away with wash buffer. Substrates (A & B) are used to visualize the HRP enzymatic reaction. HRP catalyzes thermoscientific pierce solution (TMB) to produce a blue color product that changes into yellow after adding acidic stop solution. The density of yellow is proportional to the amount of Ang II in the sample captured in the plate. O.D. absorbance was read at 450 nm in a microplate reader, and the concentration of Ang II in serum and 24 hr urine was calculated. All samples were measured in duplicate to assess assay variability. The range of the assay is 40pg/ml-640pg/ml.

Sensitivity

The minimum detectable dose (MDD) of Ang II is typically less than 8.89 pg/ml.

Specificity

This assay has high sensitivity and excellent specificity for the detection of Ang II. No significant cross-reactivity or interference between Ang II and analogues was observed.

Histopathological examination of kidney tissue

Paraffin-embedded sections were made after the kidneys were fixed with 10% formalin at pH 7.4. 4 μm thin slices were made. Histological analysis was performed by using Hematoxylin and Eosin (H&E) staining at 10x and 40x magnification. H&E remains the gold standard for general tissue structure and pathology but has poor sensitivity for fibrosis detection. Masson's Trichrome is superior for detecting and quantifying fibrosis, making it the preferred stain in renal, hepatic, and cardiac fibrosis studies. Lesion scores were assigned according to the following scale: 0, no lesions; 1+, less than 25% glomerular involvement; 2+, 26% to 50%; 3+, 51% to 75%; and 4+, more than 76% [15].

## Results

Blood pressure recording

**Figure 1:**
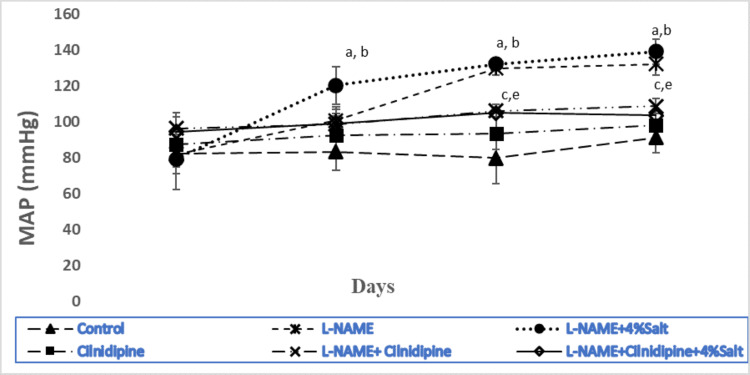
Mean arterial blood pressure with or without cilnidipine across groups The figure presents the time course of mean arterial blood pressure (MAP) during an intervention period of 28 days. One-way ANOVA test was done. Multiple group comparison was done by post hoc Tukey's multiple comparison test. Superscript a, b, c, and e indicate significant difference between groups 1, 2, 3, and 5 respectively.  *p<0.05. n=6/group. MAP was significantly increased in the experimental hypertensive group and significantly decreased with cilnidipine treatment. L-NAME: NG-nitro-L-Arginine Methyl Ester.

Blood pressure (systolic and diastolic) of conscious rats was measured weekly for four weeks. The mean arterial blood pressure at baseline did not differ significantly across all groups. Throughout the intervention period, no statistically significant variation in the mean arterial pressure of the control group was noted. A significant increase in MAP was observed in L-NAME and L-NAME plus salt-treated rats from the first week onwards (p˂0.05). MAP significantly decreased (p˂0.05) in the groups (4 & 6) receiving simultaneous cilnidipine treatment (Figure 1).

Excretion of urinary protein and creatinine

In experimental hypertensive rats (groups 3 and 5 rats), we observed a significant increase in proteinuria and a significant reduction in creatinine clearance (Table 1). Concurrent treatment with cilnidipine (group 4 and 6 rats) resulted in a significant decrease in proteinuria and a significant increase in creatinine clearance (p˂0.05).

**Table 1:** Proteinuria and creatinine clearance across groups Superscripts a, b, c, d, and e indicate significant difference between groups. *Statistically significant if *p≤0.05.  Urinary protein (24hr) in mg/ml and creatinine clearance per gm of kidney tissue in (ml /min) were significantly different between groups.  “-” fold changes are calculated versus control, hence there is no value for control. L-NAME: NG-nitro-L-Arginine Methyl Ester.

Groups (n=6/group)		24 hr urinary protein (mg/ml) Mean ± SD (95% confidence intervals)	24 hr urinary protein fold changes vs control	Creatinine clearance/gm of kidney tissue in (ml /min) Mean ± SD (95% Confidence Intervals)	Creatinine clearance fold changes vs control
Control		1.56±0.044(1.521-1.612)	-	0.078±0.0044(0.073-0.0827)	-
Cilnidipine (Cil)		1.66±0.099(1.556-1.763)	0.064102564	0.079±0.004(0.0755-0.081)	0.01282051
L-NAME		4.58±0.27^a,b^(4.289-4.881)	1.935897436	0.06±0.0019^a,b^(0.0587-0.0626)	0.23076923
L-NAME + Cil		3.38±0.24^a.b,c^(3.135-3.625)	1.166666667	0.077±0.0027^c^(0.0743-0.0797)	0.01282051
L-NAME + Salt		4.65±0.29 ^a,b,d^ (4.349-4.460)	1.980769231	0.063±0.0026 ^a,b,d ^(0.0603-0.0657)	0.19230769
L-NAME + Salt + Cil		3.08±0.18 ^a,b,c,e ^(2.899-3.264)	0.974358974	0.076±0.004 ^c,e^ (0.0728-0.0792)	0.02564103
ANOVA	F value	251.79		44.421	
	P value	0.0001*		0.0001*	

Serum urea and creatinine levels

We observed that the serum urea levels in each of the six groups did not significantly alter. We found that group 3 and 5 hypertensive rats had much higher serum creatinine levels (0.611 ±0.04) compared to the control (0.40±0.04) and that these levels significantly decreased when L-NAME and cilnidipine (0.50±0.04) were given concurrently (p˂0.05). (Table 2). Even if renal clearance is mildly affected, the high permeability of urea across membranes maintains relatively stable serum levels. In hypertension, renal medullary blood flow decreases, activating vasopressin-mediated urea reabsorption. This can compensate for reduced urea clearance, keeping serum levels stable despite kidney dysfunction [16].

**Table 2:** Estimation of serum urea and creatinine across groups Superscripts a, b, c, d, and e indicate significant difference between groups. *Statistically significant if *p≤0.05. Serum urea (mg/dl) and creatinine (mg/dl) were significantly different between groups. “-” fold changes are calculated versus control, hence there is no value for control. L-NAME: NG-nitro-L-Arginine Methyl Ester.

Groups (n=6/ group)		Serum urea (mg/dl) Mean±SD (95% Confidence Intervals)	Serum urea fold changes vs control	Serum creatinine (mg/ml) Mean±SD (95%confidence intervals)	Serum creatinine fold changes vs control
Control		42.37±2.73(38.51-44.23)	-	0.40±0.04 (0.36-0.44)	-
Cilnidipine(Cil)		43.23±3.27(39.70-46.55)	0.019829375	0.52±0.05^a ^(0.47-0.56)	0.3
L-NAME		44.02±2.93(40.94-47.07)	0.038044731	0.611±0.04^a,b ^(0.57-0.65)	0.5275
L-NAME + Cil		41.00±3.35(37.49-44.51)	-0.031588656	0.40±0.04^b,c ^(0.36-0.43)	0
L-NAME + Salt		52.35±3.09 ^a. b, c, d^ (49.11 -55.58)	0.230112981	0.50±0.04 ^a,c,d^ (0.46-0.53)	0.25
L-NAME + Salt + Cil		47.64±4.7 ^d ^(42.80-52.47)	0.121512566	0.50±0.06^c,d ^(0.45-0.56)	0.25
ANOVA	F value	9.927		23.69	
	P value	0.0001*		0.0001*	

Quantitative estimation of urinary and serum angiotensin II by ELISA

Table 3 depicts that Ang II levels were significantly increased (1.99 fold vs control) in serum and kidney tissue in L- L-NAME plus salt-treated rats compared to control. With cilnidipine supplementation, there is a significant decrease (1.99 to 1.84fold) in serum Ang II and kidney tissue Ang II levels (p˂0.05). 

**Table 3:** Comparison of serum and urinary Ang II levels across groups Superscripts a, b, c, d, and e indicate significant differences between groups. Values are statistically significant if *p<0.05. Serum Ang II (pg/ml), and urinary Ang II (pg/ml) were significantly different between groups.  “-” fold changes are calculated versus control; hence there is no value for control. Cil: Cilnidipine; Ang II: Angiotensin II; L-NAME: NG-nitro-L-Arginine Methyl Ester

Groups(n=6rats)		Serum Ang II (pg/ml) Mean ±SD (95% Confidence Intervals)	Serum Ang II Fold Changes vs control	Urinary Ang II (pg/ml) Mean ±SD (95%confidence intervals)	Urinary Ang II Fold Changes vs control
Control		152.53±25.23 (126.06-179.01)	-	330.74±7.14 (322.25-337.22	-
Cilnidipine(Cil)		161.12±42.59 (164.42-253.82)	0.056	321.10±4.59(399.18-408.80)	-0.024
L-NAME		321.61±25.87^a,b ^(294.47-348.75)	1.108	450.84±10.31^a,b ^(440.01-461.65)	0.367
L-NAME + Cil		410.49±6.9^a,b,c ^(230.11-260.80)	1.691	410.49±6.9^a,b,c^ (403.34-417.64)	0.245
L-NAME + Salt		457.42±9.29^a,b,d ^(350.41-420.53)	1.998	457.42±9.29^a,b,d^ (437.67-457.16)	0.387
L-NAME + Salt + Cil		433.77±11.3^a,b,c,e ^(235.47-280.30)	1.843	433.77±11.3^a,b,c,e ^(422.01-445.52	0.315
ANOVA	F value	49.70		166.12	
	P value	0.0001*		0.0001*	

Relative expression of serum ACE protein

**Figure 2:**
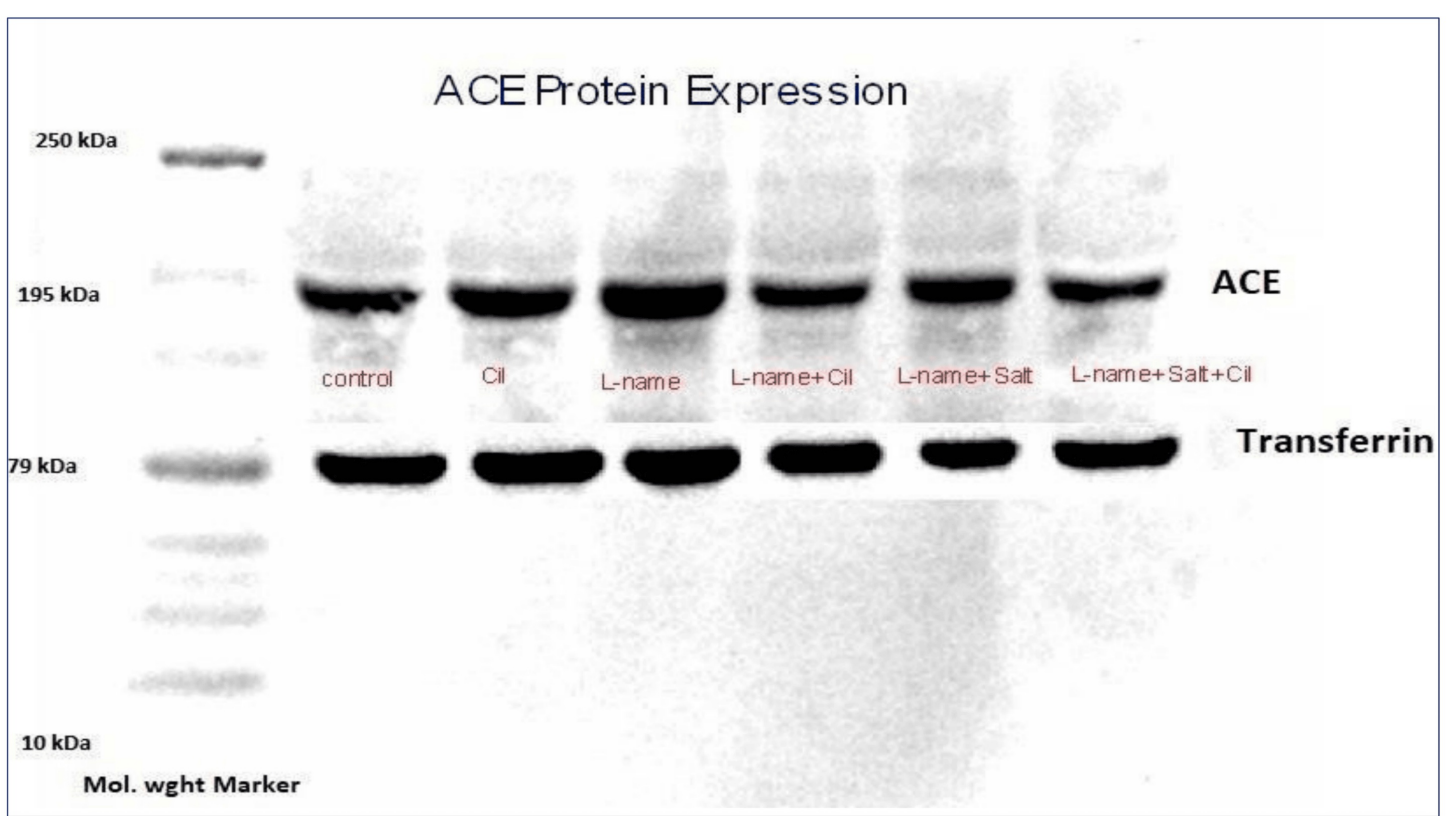
Serum ACE protein expression across groups The figure depicts ACE protein expression in serum by western blotting. We observed that there is relatively more expression of ACE protein in L-NAME and L-NAME plus salt-treated rats compared to the control. There is less expression of ACE protein in cilnidipine-supplemented rats.

Qualitative estimation of ACE protein was done by western blotting. We observed relatively more expression of ACE protein in L-NAME and L-NAME plus salt-treated rats compared to the control. There is less expression of ACE protein in cilnidipine-supplemented rats (Figure 2).

Relative expression of kidney tissue ACE protein

**Figure 3:**
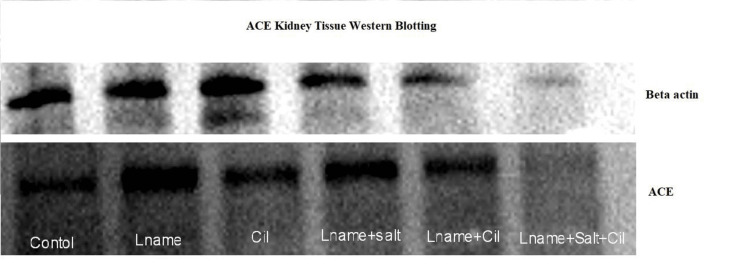
Relative expression of ACE protein in kidney tissue among groups The figure shows ACE protein expression in kidney tissue among experimental groups. We observed that there is more expression of ACE protein in L-NAME and L-NAME plus salt-treated rats compared to the control. On supplementation with cilnidipine, there is relatively less expression of ACE protein.

Qualitative estimation of ACE protein was done by western blotting. We observed that there is more expression of kidney tissue ACE protein in L-NAME and L-NAME plus salt-treated rats than in the control group. In supplementation with cilnidipine, there is relatively less expression of ACE protein (Figure 3).

Histological study of kidneys

**Figure 4:**
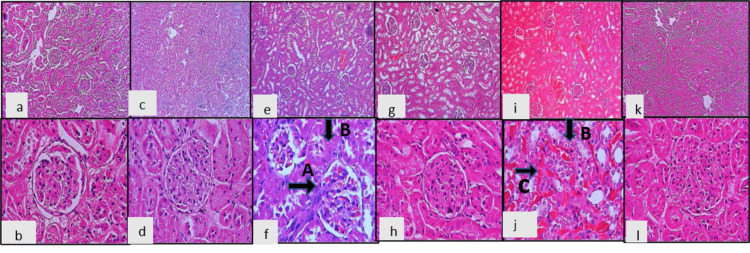
Histopathological examination of kidney tissue across groups (a)(x10): (b) (x40):control; (c) (x10): (d)) (x40): Cilnidipine; (e) (x10): (f) (x40): L-NAME;  (g) (x10): (h) (x40): L-NAME plus Cil; (i) (x10): (j) (x40): L-NAME plus Salt; (k) (x10): (l) (x40): L-NAME plus Salt and Cil.  Arrow A: Hypercellular glomeruli with increased mesangial proliferation. Arrow B: Focal epithelial hydropic degeneration of renal tubules. Arrow C: Interstitial congestion. L-NAME - NG-nitro-L-Arginine Methyl Ester; Cil: cilnidipine.

Renal tissue microscopic structure is shown in two rows using hematoxylin and eosin (10 x & 40x) staining. Normal renal histopathology was seen in the control and cilnidipine groups. We observed that the L-NAME plus 4%NaCl-treated rats' glomeruli are hypercellular and have increased mesangial proliferation (arrow A). Tubular epithelium shows focal hydropic degeneration (arrow B). We found almost normal renal histopathology with cilnidipine treatment (Figure 4).

Mesangial cells provide structural support to the glomerular capillaries and help regulate filtration by modulating capillary surface area. Excessive mesangial proliferation leads to glomerulosclerosis, capillary occlusion, and reduced glomerular filtration rate (GFR). A reduction in proliferation slows disease progression. Studies on cilnidipine vs. amlodipine in hypertensive nephropathy show that cilnidipine reduces mesangial proliferation, leading to better long-term renal preservation [17].

Hypertensive renal injury leads to hydropic degeneration which is characterized by swelling of renal tubular cells due to intracellular water accumulation. Persistent hydropic degeneration impairs reabsorption, leading to electrolyte imbalances and progressive nephron loss, and contributes to interstitial fibrosis, a key predictor of chronic kidney disease (CKD) progression [18].

## Discussion

We found that by treatment with cilnidipine, there is an improvement in creatinine clearance, decreased glomerular damage, and decreased proteinuria and urinary Ang II levels in experimental hypertensive rats.

These actions of cilnidipine are probably mediated by its sympathetic N-type calcium channel-blocking actions in addition to the vascular L-type calcium channels. N-type calcium channels are located on the sympathetic nerve terminals. Cilnidipine, by blocking N-type calcium channels, reduces sympathetic neurotransmission, consequently lowering the enhanced sympathetic drive in hypertensive rats [15].

By blocking N-type calcium channels in sympathetic nerve terminals and the adrenal glands, cilnidipine reduces sympathetic nerve activity, leading to decreased renin release from the juxtaglomerular apparatus. This results in lower aldosterone levels, reducing sodium and water retention. In contrast, conventional L-type CCBs may activate the RAAS as a compensatory mechanism, leading to reflex sodium retention and edema [19].

We measured the relative expression of ACE in experimental hypertensive rats with or without cilnidipine in order to assess its impact on the RAS. The expression of the ACE protein in serum and kidney tissue significantly decreased with cilnidipine treatment (Figures 2A, 2B). Toba et al. showed that in hypertensive rats, cilnidipine reduced the expression of the ACE gene in the kidney, but not amlodipine. They propose that cilnidipine, by blocking N-type calcium channels, would limit sympathetic nerve activity and protect the kidney [3]. The inhibition of ACE not only reduces Ang II levels but also increases bradykinin availability. Bradykinin is a potent vasodilator and promotes natriuresis by enhancing renal blood flow and reducing sodium reabsorption. Bradykinin counteracts the RAS by reducing aldosterone synthesis [20].

We also observed a significant decrease in serum and urinary Ang II levels with cilnidipine treatment. The primary bioactive peptide of the RAS, angiotensin-II, is crucial for controlling the structure and functions of blood vessels. Strong vasoconstrictor angiotensin-II can stimulate sympathetic nervous system activity. In a nitric oxide deficient hypertensive rat model, sympathetic hyperactivity can result in vascular remodeling, heart failure, and RAAS imbalance [21]. Cilnidipine reduces the production of angiotensin II by reducing renin release and the activity of the ACE. Additionally, in the spontaneously hypertensive rats, it might reduce the renal expression of angiotensinogen [22]. These findings suggest that N-type calcium channels play a significant role in modulating renin-angiotensin system activity.

Reduction in Ang II levels leads to upregulation of AT₂ (angiotensin II) receptors. Enhanced AT₂ receptor signaling promotes vasodilation, reduced fibrosis, and renal protection. This effect is beneficial in diabetic nephropathy, hypertensive nephropathy, and heart failure. With reduced Ang II, more angiotensin I (Ang I) is diverted toward Ang-(1-7) synthesis. Ang-(1-7) binds to the Mas receptor, exerting vasodilatory, anti-inflammatory, and anti-fibrotic effects [23]. ACE2 activation or recombinant Ang-(1-7) therapy is being explored as a novel approach for cardiovascular and renal protection.

We found a significant decrease in proteinuria with cilnidipine treatment, similar to our findings. A clinical investigation comparing the cilnidipine (both L/N- type CCB) and amlodipine (only L-type CCB) revealed that cilnidipine is significantly more effective than other CCBs at stopping the excessive urinary protein excretion in hypertensive individuals. Additionally, they demonstrated that cilnidipine was more effective in protecting the glomerular membrane and avoiding kidney impairment compared to blockers of only L-type Calcium channels. They conclude that suppression of RAS hyperactivity by cilnidipine can be due to its sympatholytic action [24].

A clinical trial, known as the Cilnidipine versus Amlodipine Randomized Trial for Evaluation in Renal Disease (CARTER), demonstrated that cilnidipine, when added to renin-angiotensin system inhibitors, significantly reduced proteinuria in hypertensive patients with chronic kidney disease compared to amlodipine [25]. By decreasing sympathetic discharge, cilnidipine prevents glomerular hypertension. Intra glomerular hypertension results from dilatation of only afferent glomerular arterioles. However, because cilnidipine can dilate both afferent and efferent arterioles, it prevents glomerular hypertension and protects the glomeruli and podocytes [6]. Cilnidipine is a more effective antihypertensive and reno-protective medication than amlodipine, according to a study conducted on proteinuria patients [26]. Cilnidipine and olmesartan both have the ability to lower blood pressure in diabetic hypertensives, even though by different mechanisms, micro-albuminuria was more efficiently reduced by cilnidipine than olmesartan [27].

We found reduced mesangial proliferation and focal hydropic degeneration of tubular epithelium on histopathological study of kidney tissue. Excessive mesangial proliferation leads to glomerulosclerosis, capillary occlusion, and reduced GFR. A reduction in proliferation slows disease progression. Studies on cilnidipine vs. amlodipine in hypertensive nephropathy show that cilnidipine reduces mesangial proliferation, leading to better long-term renal preservation [28]. Hypertensive renal injury leads to Hydropic degeneration, which is characterized by swelling of renal tubular cells due to intracellular water accumulation. Persistent hydropic degeneration impairs reabsorption, leading to electrolyte imbalances, progressive nephron loss, and contributing to interstitial fibrosis, a key predictor of CKD progression. In models of hypertensive nephropathy, cilnidipine has been shown to reduce tubular injury and hydropic degeneration, suggesting its protective effect on renal tubules [3].

Cilnidipine has been reported to have more beneficial effects on proteinuria progression in hypertensive patients than Amlodipine, an L-type CCB. The N-type calcium channel blockade that inhibits renal sympathetic nerve activity might reduce glomerular hypertension. However, the precise mechanism of the renoprotective effect of cilnidipine remains unknown. Because cilnidipine exerted significantly higher antioxidant activity than Amlodipine in cultured human mesangial cells, it can be hypothesized that cilnidipine might exert a renoprotective effect by suppressing oxidative stress. The urinary albumin, 8-hydroxy-2'-deoxyguanosine (OHdG), a marker of oxidative stress, was significantly decreased with cilnidipine compared with those with amlodipine [29]. Thus, cilnidipine probably exerts a greater renoprotective effect through its antioxidative properties.

In our study, cilnidipine demonstrated significant renal protective effects by reducing proteinuria and improving creatinine clearance in hypertensive rats. These effects of cilnidipine may be because of its N-type calcium channel blocking actions. Cilnidipine, by blocking N-type calcium channels, plays a significant role in modulating RAS and sympathetic activity. Hence, cilnidipine may act as a renal protective agent in hypertensive patients with renal damage. However, additional large-cohort and longer-term studies will be needed to clarify whether cilnidipine is superior to other CCBs in maintaining renal function.

Future directions

Future studies involving longer treatment durations, additional endpoints (e.g., markers of fibrosis), or translational studies in humans can be done.

Limitations

While L-NAME-induced hypertension is a useful experimental tool for studying the role of NO in blood pressure regulation, its relevance to human hypertension, especially in the context of essential and secondary hypertension, is limited. The acute nature of the model, species-specific differences, and lack of a comprehensive mechanism make it less directly applicable to chronic human conditions. More complex models that better reflect the multifactorial and chronic nature of human hypertension are needed for more accurate translation of findings. Our study did not include the molecular marker of oxidative stress (e.g., 8-hydroxy-20-deoxyguanosine (8-OHdG) or MDA) and glomerular damage (liver-type fatty acid-binding protein (L-FABP).

## Conclusions

We conclude that in nitric oxide-deficient hypertensive rats, cilnidipine prevents kidney damage by improving creatinine clearance, lowering proteinuria, and preventing glomerular sclerosis, possibly by lowering oxidative stress and suppressing the RAS system by blocking N-type calcium channels. As cilnidipine may manage both blood pressure and proteinuria, it can be preferred over other L-type CCBs, particularly in patients with chronic renal disease. Cilnidipine may further prevent chronic renal disease progression. These findings suggest that cilnidipine, an L-type and N-type CCB, could be a more effective treatment for hypertensive patients with long-term renal complications. Further mechanistic studies using selective N-type channel blockers or knockout mice are needed to prove the findings.
